# Taking the Wheel – *de novo* DNA Methylation as a Driving Force of Plant Embryonic Development

**DOI:** 10.3389/fpls.2021.764999

**Published:** 2021-10-29

**Authors:** Lucija Markulin, Andreja Škiljaica, Mirta Tokić, Mateja Jagić, Tamara Vuk, Nataša Bauer, Dunja Leljak Levanić

**Affiliations:** Division of Molecular Biology, Department of Biology, Faculty of Science, University of Zagreb, Zagreb, Croatia

**Keywords:** DNA methylation, RdDM, plant embryogenesis, zygotic embryogenesis, somatic embryogenesis, RNA polymerase V, *Arabidopsis thaliana*

## Abstract

During plant embryogenesis, regardless of whether it begins with a fertilized egg cell (zygotic embryogenesis) or an induced somatic cell (somatic embryogenesis), significant epigenetic reprogramming occurs with the purpose of parental or vegetative transcript silencing and establishment of a next-generation epigenetic patterning. To ensure genome stability of a developing embryo, large-scale transposon silencing occurs by an RNA-directed DNA methylation (RdDM) pathway, which introduces methylation patterns *de novo* and as such potentially serves as a global mechanism of transcription control during developmental transitions. RdDM is controlled by a two-armed mechanism based around the activity of two RNA polymerases. While PolIV produces siRNAs accompanied by protein complexes comprising the methylation machinery, PolV produces lncRNA which guides the methylation machinery toward specific genomic locations. Recently, RdDM has been proposed as a dominant methylation mechanism during gamete formation and early embryo development in *Arabidopsis thaliana*, overshadowing all other methylation mechanisms. Here, we bring an overview of current knowledge about different roles of DNA methylation with emphasis on RdDM during plant zygotic and somatic embryogenesis. Based on published chromatin immunoprecipitation data on PolV binding sites within the *A. thaliana* genome, we uncover groups of auxin metabolism, reproductive development and embryogenesis-related genes, and discuss possible roles of RdDM at the onset of early embryonic development via targeted methylation at sites involved in different embryogenesis-related developmental mechanisms.

## Introduction

In vascular plants, embryogenesis begins by establishing cell embryogenic competence, which is followed by formation of distinct embryonic stages. Besides the dominant form of embryogenesis which involves a fertilized egg cell or a zygote (zygotic embryogenesis, ZE), flowering plants have evolved alternative fertilization-independent mechanisms of embryo formation (classified under the umbrella term asexual embryogenesis; AE). The general characteristic of AE mechanisms is the variability of cells that can develop competency for embryogenesis. Common forms of AE that occur *in vivo* are parthenogenesis, where a reduced egg cell develops the embryo, gametic embryogenesis, where an unreduced egg cell or sperm cell develops the embryo and adventitious embryony, where embryo is formed from cells of nucellus or integument (Hand et al., 2016). The rarest naturally occurring AE process is somatic embryogenesis (SE), characterized by the possibility of embryo formation from virtually any somatic cell. This process is independent not only of fertilization but also of existence of gametes, gametophyte, ovules or reproductive tissues, and is the strongest evidence of plant cell totipotency. It is thought that a plant cell in any developmental stage or form has the potential to, under suitable environmental conditions, initiate regulatory mechanisms which will lead to cell dedifferentiation to a state of competency followed by re-differentiation and consequently embryonic development (Fehér, 2005).

Although ZE and SE differ in the initiation stage of embryogenesis, evidence shows overall similarity between the two processes on the level of both morphology and genetics. For instance, a somatic cell undergoing embryogenesis mimics the zygotic pattern of cell division – in other words, just like its zygotic counterpart, it divides asymmetrically (Dodeman et al., 1997; Vasilenko et al., 2000) and forms a suspensor-like structure and a somatic embryo (Leljak-Levanić et al., 2015). Furthermore, similar to zygotic embryogenesis which is marked by existence of embryo and non-embryonic endosperm, different cell types were found in SE cultures, such as embryonic and non-embryonic cell clusters identified in maize microspore cultures (Massonneau et al., 2005). Analyses of cellular types and secreted molecules of *in vitro* cultures suggest endosperm-like functions of these non-embryonic cell clusters, which are thought to communicate with embryonic cells via signaling molecules to direct embryo development, much like the mutually dependent development of embryo and endosperm within the female gametophyte (reviewed in Matthys-Rochon, 2005). In Arabidopsis and other dicots, cultured somatic embryos go through all the major stages of development described for zygotic embryos, namely the globular, heart, torpedo and cotyledonary stage (Kurczyńska et al., 2007). Additionally, similar sets of transcription factors are active during SE and ZE, indicating similar transcriptional regulatory mechanisms between the two processes (Gliwicka et al., 2013; Jin et al., 2014; Leljak-Levanić et al., 2015). With this in mind, a recent RNAseq study of an Arabidopsis embryonic culture reveals surprising results – remarkably, the SE transcriptome has more similarities with transcriptome of germinating seeds than early zygotic embryos (Hofmann et al., 2019). Contrary to previous indications, this finding suggests there might be no general regulatory mechanisms mediating ZE and SE, but does not exclude a subset of specific mechanisms common for both ZE and SE. Identification of these specific yet common mechanisms presents both a challenge and an opportunity for implementing novel approaches to DNA methylation research. Comparative analysis of ZE and SE transcriptome during the initiation stage still holds potential for identification of a specific set of common regulators and regulatory mechanisms between the two types of embryogenesis. If we consider the vast array of possibilities that might lead to SE (different cell types, different environmental conditions etc.), it seems even more likely that some of the mechanisms and molecules involved in SE will overlap with initiation of ZE.

Embryogenesis implies a state of intensive developmental transitions. The role of epigenetic mechanisms during the initiation and maturation stages of embryogenesis was shown in analyses of mostly SE in species such as barley, soybean, common bean, cotton, Norway spruce (for a review, see Nic-Can and De la Peña, 2014), Arabidopsis (Grzybkowska et al., 2018), carrot (LoSchiavo et al., 1989; Yamamoto et al., 2005), pumpkin (Leljak-Levanić et al., 2004) and others. In Arabidopsis, DNA methylation mechanisms have been shown to underlie both ZE (Xiao et al., 2006; Pillot et al., 2010; Ingouff et al., 2017; Forgione et al., 2019) and SE (Grzybkowska et al., 2018; Osorio-Montalvo et al., 2018). Here, we review recent findings on DNA methylation during plant ZE and SE, and propose a central role of RdDM in gene expression regulation during these processes. Assuming that RdDM activity is determined by PolV targeting, we analyze recently published chromatin-immunoprecipitation data based on the Arabidopsis genome (Liu et al., 2018) and list genes related to reproductive development, embryogenesis and auxin dynamics as possible targets of RdDM.

## Epigenetic Reprogramming and DNA Methylation in Early Plant Development

DNA methylation is an epigenetic mechanism commonly found in mammals, plants, filamentous fungi, fish and insect species, among others (Martienssen and Colot, 2001; Li, 2002; Chan et al., 2005; Zhong, 2016; Bewick et al., 2017; Anastasiadi et al., 2018). While many aspects of DNA methylation show striking levels of evolutionary conservation, different organisms have also evolved unique mechanisms. For instance, despite a high structural similarity between mammal and plant methyltransferases, the exact mechanisms by which they establish DNA methylation and the regulatory factors they associate with during this process are often different (Zhong, 2016). In contrast to mammals that primarily methylate CG dinucleotides, plants methylate their DNA in all sequence contexts: symmetric CG, CHG, and asymmetric CHH (H = A, C, or T) by different classes of DNA methyltransferases (Elhamamsy, 2016).

Pioneer work in the field has associated DNA methylation with a range of cellular functions, including transposable element silencing, maintenance of genome integrity, genomic imprinting and X-chromosome inactivation (for a review, see Zhang et al., 2018). In recent years, the focus of attention has become the elucidation of DNA methylation mechanisms in regulation of gene expression, which has also been implicated during plant growth and development (Finnegan et al., 1996; Jacobsen et al., 2000; Xiao et al., 2006; Bartels et al., 2018; Zhang et al., 2018).

In plants, global methylation levels are dynamic and variable throughout development. On the one hand, DNA methylation can be conservatively inherited through cell divisions, ensuring epigenetic memory of their cellular predecessors and can be heritable across generations (Schmitz et al., 2013; Iwasaki and Paszkowski, 2014). On the other hand, differences in methylation profiles can be found even between cells of the same origin separated by only a few divisions, such as different cells of a plant gametophyte, as shown for Arabidopsis and rice (Ibarra et al., 2012; Park et al., 2016; Han et al., 2019; Borg et al., 2021). Perhaps the most dramatic feature of epigenetics is ‘epigenetic reprogramming,’ a term used to describe a process in which epigenetic marks of a previous developmental stage or cellular form are erased and a novel epigenetic pattern is established *de novo*. Plants are remarkable in this aspect because they seem to possess a dual ability to both stably inherit epialleles across generations, and to undergo significant epigenetic reprogramming during male and female gametophyte development and embryogenesis (reviewed in Kawashima and Berger, 2014; Gehring, 2019). In recent years, several papers demonstrated the occurrence of epigenetic reprogramming during developmental transitions in different species of the plant kingdom. In the liverwort *Marchantia polymorpha*, a species with a dominant gametophyte generation, epigenetic reprogramming occurs at least twice, once in the gametophytic and once in the sporophytic generation (Schmid et al., 2018). Because the morphology and transcriptional profiles of flowering plants markedly shift between the haploid gametophyte and diploid sporophyte, it is safe to assume that epigenetic reprogramming occurs here as well, once at the diploid-to-haploid transition and a second time during haploid-to-diplod transition. In Arabidopsis, the loss of histone H3 methylation (H3K9me2) and DNA demethylation of transposon-associated *cis*-regulatory elements guides the diploid-to-haploid transition, which later in the vegetative nucleus of pollen grain unlocks genes involved in sperm cell transport and delivery. Conversely, the loss of another methylation mark (H3K27me3) underlies the haploid-to-diploid transition in sperm cells, unlocking the set of developmental genes required to initiate development of the new generation upon fertilization (Borg et al., 2020, 2021). Similar epigenetic reprogramming might regulate egg and central cell fates and transitions between haploid and diploid generations in the female gametophyte. Furthermore, it seems plausible that embryonic epigenetic reprogramming is involved in control of post-embryogenic development, as specifically shown for a seed-specific transcription factor in Arabidopsis (Tao et al., 2017), and that epigenetically based communication pathways exist between distinct embryonic stages to finely tune development of a new organism.

### DNA Demethylases in Plants

In plants, as in mammals, the loss of DNA methylation marks can be achieved passively during cell division when DNA methyltransferases are inactive, but it can also be an active, site-specific process (Furner and Matzke, 2011; Elhamamsy, 2016). In mammals, active demethylation occurs by oxidation or deamination. First, ten–eleven translocation enzymes (TET) hydroxylate 5-methylcytosine to 5-hydroxymethylcytosine. Further oxidation by TET produces 5-formylcytosine, which can be either further oxidized or cleaved by thymine-DNA glycosylase (TDG) (Elhamamsy, 2016). In plants, DEMETER DNA GLYCOSYLASES (DME) and REPRESSOR OF SILENCING 1 (ROS1) are multifunctional enzymes that function as DNA gylcosylases that specifically excise 5-methylcytosine through cleavage of the *N*-glycosylic bond (Penterman et al., 2007).

ROS1 is the dominant DNA demethylase in vegetative tissues (Gong et al., 2002), where it presumably targets specific TEs and prevents spreading of their methylation patterns onto nearby protein-coding genes (Tang et al., 2016). In reproductive tissues, DME is the major DNA demethylase specifically expressed in the central cell of the female gametophyte, i.e., the future endosperm (Choi et al., 2002) and the vegetative cell of the bicellular male gametophyte (Schoft et al., 2011). In the endosperm, DME is involved in establishing gene imprinting, or the preferential expression of either the maternal or paternal allele of the same gene. For instance, DME demethylates Polycomb-group protein genes *MEDEA* (*MEA*) and *FERTILIZATION INDEPENDENT SEED 2* (*FIS2*) (Gehring et al., 2006; Jullien et al., 2012) and a transcription factor gene *FLOWERING WAGENINGEN* (*FWA*) (Kinoshita et al., 2004), all of which are maternally expressed. The exact mechanism of gene imprinting regulation is still unclear, with indications of several additional factors other than DME affecting endosperm imprinting, such as the antagonistic effect of DNA methylation (Xiao et al., 2003), histone methylation (Gehring et al., 2006), and parental genome dosage imbalance (Jullien and Berger, 2010). Nevertheless, the importance of DME during Arabidopsis reproductive development is illustrated by evidence that DME accounts for all demethylation in the central cell (Choi et al., 2002), and that central cell demethylation also reinforces transposon methylation in the egg cell (Park et al., 2020). The same scenario occurs in the male gametophyte, all of which probably contributes to stable silencing of transposable elements across generations (Ibarra et al., 2012). Functionally related to DME and ROS1 demethylases, proteins known as Effector of transcription (ET) were recently proposed as epigenetic regulators during reproductive development. Lack of ETs expression is manifested during gametophyte and endosperm development (Tedeschi et al., 2019), suggesting them as novel plant-specific regulators of DNA methylation during reproduction.

### Maintenance and *de novo* Methyltransferases in Plants

DNA methylation can be either maintained or established *de novo*. In plants, two DNA methyltransferases work to maintain DNA methylation, DNA METHYLTRANSFERASE 1 (MET1), an ortholog of mammalian DNMT1 which maintains CG methylation, and the plant-specific CHROMOMETHYLASE 3 (CMT3) which maintains CHG methylation (H = A, C, or T) (Finnegan and Kovac, 2000; Chan et al., 2005). A related methyltrasferase, CMT2, maintains CHG and CHH methylation in a process guided by methylation of histone H3 (Stroud et al., 2014). A different pathway, RNA-directed DNA methylation (RdDM) is responsible for *de novo* DNA methylation in all three sequence contexts and is mediated by activity of two methyltransferases, DOMAINS REARRANGED METHYLTRANSFERASE 1 and 2 (DRM1 and DRM2) (Cao and Jacobsen, 2002; Zhang and Jacobsen, 2006). RdDM is controlled by a two-armed mechanism based around the activity of two RNA polymerases. PolIV transcribes siRNA precursors (P4-RNAs), which are processed in two steps: first, RNA-DEPENDENT RNA POLYMERASE 2 (RDR2) transcribes them into double-stranded RNAs (Haag et al., 2012) and then the DICER-LIKE 3 (DCL3) protein cleaves them into 24 nt-long siRNAs (Qi et al., 2005). The ARGONAUTE 4 (AGO4) protein binds the siRNAs, forming AGO4-siRNA complexes (Qi et al., 2006; Kuo et al., 2017). The second polymerase, PolV, produces long non-coding RNAs (lncRNAs) using specific genomic loci as templates (Wierzbicki et al., 2008; Böhmdorfer et al., 2016). Genomic positioning of PolV is reinforced through binding of previously methylated DNA sites by the SU(VAR)3–9 homolog proteins SUVH2 and SUVH9 (Liu et al., 2014) and interaction with the DDR complex consisting of DEFECTIVE IN MERISTEM SILENCING 3 (DMS3), DEFECTIVE IN RNA-DIRECTED DNA METHYLATION 1 (DRD1), and RNA-DIRECTED DNA METHYLATION 1 (RDM1) (Zhong et al., 2012). It is thought that PolV-produced lncRNAs act as scaffolds for base-pairing with siRNA and associated AGO4 (Wierzbicki et al., 2009) which brings the main components of the two arms of RdDM – one led by PolIV and the other by PolV – into contact with the DRM2-mediated methylation machinery, recruiting it onto specific sites on the genome (Zhong et al., 2014). The mechanism described is the so-called canonical RdDM pathway and according to its current model, the genomic position destined for methylation is determined primarily by the activity of PolV and its suite of supporting proteins (Zhong et al., 2012; Böhmdorfer et al., 2016). Novel findings constantly challenge the current model of RdDM. For instance, although the model assumes that the slicing activity of AGO4 is not required for siRNA biogenesis, recent evidence shows that a subset of 24 nt-siRNAs is indeed sliced by AGO4, which possibly occurs in a self-reinforced loop dependent on PolV and DRM2 (Wang and Axtell, 2017). Not only that, AGO4 can also slice PolV nascent transcripts, suggesting a dual mechanism by which AGO4 recruits DRM2 through both protein-protein interaction (current model) and Pol V transcript slicing (Liu et al., 2018). The importance of AGO4 and related AGO6 and AGO9 was highlighted in a study by Gallego-Bartolomé et al. (2019) who analyzed the order of action within the RdDM pathway and the ability of different components to induce methylation when others are mutated. Their results show an essential role of AGO proteins in methylation targeting and, to make matters even more complex, show that an AGO protein can successfully bridge PolV and DRM2 to induce *de novo* DNA methylation even in the absence of siRNAs produced by PolIV (Gallego-Bartolomé et al., 2019). There is still a long way to go in understanding the mechanisms and roles of RdDM in plants. Indeed, canonical RdDM further extends into several non-canonical pathways which, like canonical RdDM, utilize siRNA-AGO-PolV complexes, but in which siRNAs are produced by Pol II. Non-canonical RdDM pathways are largely unexplored, possibly due to their minor role in transcription silencing. They are limited in their dependence on Pol II production of mRNA and are mostly targeting the same loci as canonical RdDM, seemingly acting as a means to produce alternatively sourced siRNAs to feed into the more predominant canonical form (for a review, see Cuerda-Gil and Slotkin, 2016). Canonical or not, there seems to be a consensus about the crucial role of PolV in determining the genomic site to be methylated via RdDM. This is particularly interesting in the context of land plant evolution – unlike the PolIV arm of RdDM, which is commonly found in land plant species, the PolV arm has reached its most complex level in flowering plants, involving several plant-specific members, and characterized by rapid evolution of its main polymerase (for a review, see Matzke et al., 2015).

**FIGURE 1:**
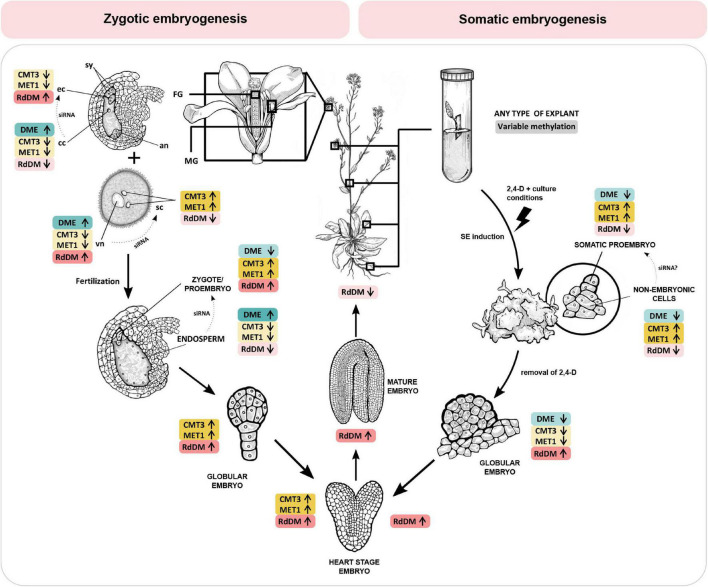
DNA methylation mechanisms change activity during specific stages of zygotic and somatic embryogenesis in *Arabidopsis thaliana*. CMT3 and MET1 (yellow) maintain DNA methylation in CHG and CG context, respectively. The RdDM pathway (red) methylates DNA *de novo* in all contexts, and DME (blue) actively demethylates DNA regardless of context. Zygotic embryogenesis **(left)**. Before fertilization, RdDM is the dominant DNA methylation mechanism in the egg cell. Conversely, in the central cell, CMT3, MET1 and RdDM activity is low and DME activity is high, resulting in DNA hypomethylation. In the two spermal cells, CMT3 and MET1 are the dominant methyltransferases. Conversely, in the vegetative cell CMT3 and MET1 activity is low and DME activity is high, resulting in DNA hypomethylation. RdDM activity in the vegetative nucleus progressively increases. Embryogenesis begins when two spermal cells fertilize the egg and central cell of the female gametophyte, respectively. After fertilization, DNA methylation in the zygote and proembryo increases due to inherited activity of RdDM and increased expression of MET1 and CMT3. In the endosperm, all three methylation mechanisms reduce their activity, and DME remains active, resulting in DNA hypomethylation. RdDM activity progressively increases during embryo maturation, and drops after germination. Somatic embryogenesis **(right)**. Upon induction of somatic embryogenesis by 2,4-D and specific culture conditions, CMT3 and MET1 become the dominant methyltransferases in both the somatic embryo and the surrounding non-embryonic cell clusters. DME activity is low. RdDM activity is initially low but progressively increases during embryo maturation, following a course similar to zygotic embryogenesis. The DNA of the central cell, vegetative nucleus and the endosperm is hypomethylated, resulting in expression of transposons and eventually biogenesis of siRNA which are transferred into the egg cell, spermal cells and the embryo, respectively (dotted arrows), to ensure genome stability. As of yet, there is no evidence of siRNA-mediated communication between somatic proembryo and non-embryonic cells in culture. Information presented in the figure is based on findings published in Jullien et al. (2012); Grzybkowska et al. (2018), and Gehring (2019). 2,4-D, 2,4-dichlorophenoxyacetic acid; an, antipodal cells; cc, central cell; CMT3, CHROMOMETHYLASE 3; DME, DEMETER DNA GLYCOSYLASE; ec, egg cell; FG, female gametophyte; MET1, DNA METHYLTRANSFERASE 1; MG, male gametophyte; RdDM, RNA-directed DNA methylation; sc, sperm cell; siRNA, small interfering RNA; sy, synergide; vn, vegetative nucleus. Enzyme activity is indicated by arrows and color intensity (up/dark – high activity, down/bright – low activity).

In the following chapters, we discuss the role of DNA methylation during plant reproductive development and embryogenesis. Figure 1 illustrates the changes in activity of maintenance methyltransferases (CMT3 and MET1), the RdDM pathway and demethylase DME during specific developmental stages of zygotic and somatic embryogenesis in *Arabidopsis thaliana*, providing an overview of latest findings and a comparison of the two processes.

## DNA Methylation at the Onset of Zygotic Embryogenesis

Zygotic embryogenesis in flowering plants begins with the process of double fertilization. Of the two identical sperm cells, one fuses with the egg cell and the other with the central cell, which leads to simultaneous formation of the embryo and the endosperm, respectively. In other words, within the female gametophyte, in the mutually close proximity begins the rise of two distinct kinds of progeny, the embryo as the progenitor of the next generation and the triploid endosperm with a temporary and supporting role. The majority of findings on the subject of angiosperm embryogenesis was built on evidence gained from *A. thaliana*, a species with highly predictable patterns of cell division and cell fate determination during embryogenesis (Mayer et al., 1993; Möller and Weijers, 2009).

Arabidopsis embryogenesis begins with a two-fold to three-fold elongation of the zygote, followed by the first asymmetric cell division which gives rise to a two-celled proembryo. The apical cell gives rise to most of the embryo, while the basal cell forms the extraembryonic suspensor which gradually disintegrates through programmed cell death. Only the topmost cell of the suspensor, the hypophysis, comprises the embryo and later forms a root meristem (Willemsen and Scheres, 2004). From the very onset of embryogenesis, asymmetricity plays the lead role, as eventually evident by establishment of the apical-basal axis which will guide the development of shoot and root tissues later on. Elongation and asymmetric division of the zygote is coordinated by two leading factors: a paternally activated MAPKK Kinase YODA (YDA) and a patterning gene *WOX8* (Lukowitz et al., 2004; Ueda et al., 2011). The YDA signaling pathway regulates zygote elongation and induces phosphorylation of transcription factor WRKY2, which then directly activates WOX8 and leads to a polarized positioning of organelles and eventually an asymmetric zygote division (Ueda et al., 2011, 2017). The YDA-WRKY2-WOX8 signaling cascade is the first major regulatory point at which DNA methylation could affect early embryo development, and there has been indication that MET1 might play a role in this process (Figure 1). Namely, mutations of the *MET1* gene significantly impact DNA methylation, *YDA*, *WOX2* and *WOX8* gene expression, and embryo development (Xiao et al., 2006; Table 1).

**TABLE 1:** Changes in DNA methylation and/or gene expression detected in embryos and young seedlings of Arabidopsis mutants with non-functional DNA methylation mechanisms.

Mutant	Developmental stage or tissue type	Gene(s) or sequences	Methylation status	Expression status	References
*met1*	Embryo at 4 DAP	*YDA*	Not tested	UP	Xiao et al., 2006
	10 days old seedlings	*YDA*	↓ CG	Not tested	
	Embryo at 4 DAP	*WOX2, WOX8*	Not tested	DOWN	
	10 days old seedlings	*PIN1*	No mCG detected	Not tested	

*drm1*	Embryo	*MEA* (methylation marker)	= CHH	Not tested	Jullien et al., 2012

*drm2*	Embryo	*MEA* (methylation marker)	↓ CHH	Not tested	Jullien et al., 2012
	Egg cell	Globally	*↓ CHH	Not tested	Ingouff et al., 2017

*drm1 drm2*	Embryo	*MEA* (methylation marker)	*↓ CHH	Not tested	Jullien et al., 2012
	5–15 days old somatic embryo	*LEC1, LEC2, BBM*	Not tested	UP	Grzybkowska et al., 2018
	Meiocyte	*RPS16B*	↓ CG, CHG, CHH	UP	Walker et al., 2017
		AT5G67280, AT2G23430	↓ mC	UP	
		*MPS1 (PRD2)*	↓ CG, CHG, CHH	UP, mis-spliced	
	Closed flower	*SPL*/*NZZ*	Not tested	UP	Mendes et al., 2020

*drm1 drm2 cmt3*	13 days old leaves	*YUCCA2*	↓ mC	UP	Forgione et al., 2019
		*TAA1, ARF7*	↓/ = mC	UP	
		*SAUR76, PIN1, PIN3, PIN4*	Not tested	DOWN	
	6 days old roots	*PIN1*, *PIN7*	Not tested	DOWN	
	5–15 days old somatic embryo	*LEC1, LEC2, BBM*	Not tested	UP	Grzybkowska et al., 2018

*nrpd1b*	Embryo	*MEA* (methylation marker)	*↓ CHH	Not tested	Jullien et al., 2012

*nrpd1a*	Egg cell	Globally	↓/ = CHH	Not tested	Ingouff et al., 2017

*nrpd1 nrpe1*	Egg cell	Globally	↓ CHH	Not tested	Ingouff et al., 2017

*nrpd2a nrpd2b*	Embryo	*MEA* (methylation marker)	*↓ CHH	Not tested	Jullien et al., 2012

DAP, days after pollination; (↓), decreased; (*↓), significantly decreased; (↓/=), slightly decreased; (=), no significant change; mC, changes in cytosine methylation with no differentiation between sequence contexts; UP, upregulated; DOWN, downregulated.

The plant hormone auxin is the second major component guiding the establishment of the apical-basal axis. Specifically, what drives axis development is the sum effect of auxin biosynthesis, canalization and global distribution. In Arabidopsis, the bulk of indole-3-acetic acid, a predominant form of auxin, is synthesized from tryptophan in two steps. The first step is catalyzed by TRYPTOPHANE AMINOTRANSFERASE OF ARABIDOPSIS 1 (TAA1) and the TAA1-related enzymes TAR1/TAR2, and the second step is under control of YUCCA monooxygenases (YUC1–11). Expression of these genes has been interpreted as a proxy for auxin production (Zhao, 2012.) Interestingly, transcription of *YUCCA* was also shown to be methylation-dependent (Forgione et al., 2019). During embryogenesis, auxin is distributed into developmentally relevant auxin maximums via activity of embryogenic efflux carriers of the PINFORMED (PIN) family (Friml et al., 2003). Their expression is also regulated by methylation, which can be induced by different classes of methyltransferases (Xiao et al., 2006; Forgione et al., 2019). The first PIN protein expressed in the early embryo is PIN7, whose activity is limited to the basal cell after the first division of the zygote, and later the suspensor. The protein localizes in the apical domain of the plasma membrane, which results in a bottom-to-top efflux of auxin and creates an auxin maximum in the apical cell. Lack of PIN7-derived auxin maximum causes an abnormal division of the apical cell, which highlights the importance of directed auxin efflux at the 2-celled proembryo stage. In *pin7* mutant embryos, the auxin maximum shifts basally into the suspensor (Friml et al., 2003; Robert et al., 2013). A similar pattern emerges in the triple methylation mutant *drm1 drm2 cmt3*, also termed *ddc* (Forgione et al., 2019).

### Different Methyltransferases Are Dominant Before and After Fertilization

To clarify the role of DNA methylation during embryogenesis, Jullien et al. (2012) analyzed the activity of specific DNA methyltransferases in different embryonic stages of *Arabidopsis thaliana*. This study shows a dramatic shift in availability of methyltransferases between the egg cell and the zygote (Figure 1). In the egg cell, DNA methylation relies predominantly on *de novo* DNA methyltransferases DRM1 and DRM2. Expression of all three methyltranferases of the DRM class (DRM1, DRM2, and DRM3) is high, while expression of methylation-maintaining enzymes MET1 and CMT3 is low. Genes encoding other components of the RdDM pathway (AGOs, PolIV, PolV, DMS3) are also highly expressed, pointing toward an important role of RdDM during this reproductive stage (Jullien et al., 2012). Following fertilization and the first division of the zygote, *DRM1* expression dramatically decreases and DRM2 becomes the main *de novo* methyltransferase during embryogenesis (Jullien et al., 2012). This could be the cause of a significant increase in CHH methylation during embryogenesis in Arabidopsis (Bouyer et al., 2017), an effect which was also shown in soybean (Lin et al., 2017), chickpea (Rajkumar et al., 2020), and *Brassica rapa* (Chakraborty et al., 2021). Additionally, all three major DNA methyltransferases (MET1, CMT3, and DRM2) become strongly expressed in both the embryo proper and the suspensor (Jullien et al., 2012; Figure 1). The authors suggest that the fertilization event is the trigger which leads to a rise in methyltransferase activity to levels higher than those in vegetative tissues. If so, the same trend of methylation changes would be expected in both fertilized gametes, the egg and the central cell, regardless of the different levels of methylation established in them before fertilization (Gehring et al., 2009). However, fertilization of the central cell does not lead to a similar rise in DNA methyltransferase activity but actually leads to a wholly different effect – a decrease in global methylation and quantity of methyltranferases (Ibarra et al., 2012; Park et al., 2016, 2020), despite both spermal cells possessing identical regulatory potential (Ingouff et al., 2017). Therefore, strong activation of DNA methyltranferases could occur independently of fertilization and a similar rise in activity might be occurring during both ZE and SE, or any other type of asexual embryogenesis. This implies that a set of signals beyond the fertilization event marks the beginning of embryogenesis and thus shapes the methylation patterns of the early embryo, regardless of its origin.

## DNA Methylation at the Onset of Somatic Embryogenesis

Somatic embryogenesis is a process during which somatic cells gain embryogenic competence to develop morphologically distinct embryonic stages which will give rise to a new plant organism. Virtually any plant cell at any given moment has the capacity to acquire developmental characteristics of a fertilized egg cell, which is followed by intensive developmental reprogramming (Nishiwaki et al., 2000; Fehér et al., 2003; Fehér, 2005). Although SE can occur naturally, as found in the genus Kalanchoë, it is much more common in plant *in vitro* culture, where it can be induced in numerous plant species and from different types of explants if granted adequate conditions (Loyola-Vargas and Ochoa-Alejo, 2016). Acquiring embryogenic competence relies on morphological, genetic and most likely epigenetic plasticity. The first effect is dedifferentiation to a state of totipotency which can then lead to a broad spectrum of possible redifferentiation outcomes, including embryogenesis (Verdeil et al., 2007). Specific plant growth regulators or application of stressful conditions can be used to stimulate embryogenic competence in somatic cells. Auxins, and especially synthetic auxin 2,4-dichlorophenoxyacetic acid (2,4-D), are the most effective inductors of SE, while their removal from growth medium stimulates embryo maturation. Exogenous auxin helps establish the auxin gradient within the explant. The auxin maximum builds at the site of contact between medium and tissue and, following auxin uptake by the tissue, the auxin level progressively decreases depending on the direction of auxin transport within the explant. At specific sites, the optimal auxin level and hormone balance is reached, which ensures favorable conditions for acquiring embryogenic competence (Fehér, 2005). In Arabidopsis SE, much like in ZE, PIN-mediated polar transport of auxin is essential for establishing auxin gradients and subsequent induction of embryogenesis (Su et al., 2009). Similar auxin dynamics in ZE and SE are backed by similar transcription patterns of genes involved in auxin distribution and transport, as well as genes involved in regulation of specific auxin responses, such as genes encoding AUXIN RESPONSE FACTORS (ARFs) and AUXIN/IAA inhibitors (Aux/IAAs) (Gliwicka et al., 2013). In general, there are many similarities between ZE and SE at the level of gene expression. In cotton, the processes of ZE and SE share more than 50% of highly expressed genes involved in methylation, stress response, hormone response, embryonic fate regulation, polarity and pattern formation (Jin et al., 2014). A similar overlap exists in Arabidopsis, where most abundant transcription factors during SE are those involved in developmental processes, phytohormone and stress responses (Gliwicka et al., 2013) and many of these genes were also found during ZE (Leljak-Levanić et al., 2015). However, a recent global transcriptome analysis in Arabidopsis revealed a higher level of similarity between transcriptomes of SE and germinating seeds, rather than ZE, indicating more complex dynamics than suggested by previous research (Hofmann et al., 2019).

### Auxin Treatment Regulates DNA Methyltransferase Activity and Expression of Somatic Embryogenesis-Marker Genes

Reports on *Daucus carota* and Arabidopsis indicate that auxin-related conditions which promote embryogenesis are associated with DNA hypermethylation (LoSchiavo et al., 1989; Yamamoto et al., 2005; Jiang et al., 2015). Exogenous auxin increases cytosine methylation during somatic embryo induction in carrot, while auxin removal rapidly decreases it (LoSchiavo et al., 1989). This is probably a consequence of auxin-mediated increase of DNA methyltransferase gene expression and downregulation of demethylases (Figure 1), as described for Arabidopsis (Grzybkowska et al., 2018). Leljak-Levanić et al. (2004) show that in pumpkin (*Cucurbita pepo*) not only auxin treatment but other SE-inducing stress treatments, like nitrogen-starvation, cause hypermethylation of DNA during SE induction. However, in the majority of reports an inverse relationship between embryogenic competence and DNA methylation was observed. In *Eleutherococcus senticosus* (Chakrabarty et al., 2003), *Pinus nigra* (Noceda et al., 2009), and *Picea abies* (Ausin et al., 2016) DNA hypomethylation seems to be associated with early stages and embryo induction. Moreover, DNA hypomethylation provoked by demethylation agents 5-azacitide has been recommended for improving the embryogenic capacity of poorly responding plant species or for aged cultures of *Theobroma cacao* (Pila Quinga et al., 2017). Due to the diversity of results, it is clear that the global level of DNA methylation is not specifically related to the embryogenesis process but more likely reflects the epigenetic status of explants caused by tissue culture conditions.

A recent gene expression analysis of four major methyltranferases during SE in Arabidopsis shows that *MET1* and *CMT3* transcripts highly accumulate during early SE and that expression of *DRM1* and *DRM2* decreases, but is followed by a striking increase in *DRM2* expression in later stages (Grzybkowska et al., 2018). Similarly, addition of 2,4-D to carrot culture positively correlates with expression of *MET1* during induction of SE and before the formation of embryonic cell clumps (Yamamoto et al., 2005). It appears that MET1 and CMT3 are the dominant methyltransferases during induction of SE (Figure 1), and in Arabidopsis this interplay is nicely illustrated by the presence of an Auxin Response Element (AuxRE) in the *CMT3* promoter, signifying a mode through which auxin can directly control CMT3 activity (Grzybkowska et al., 2018). It is interesting to note that during Arabidopsis SE, an increase in methyltransferase gene expression is combined with a decrease in expression of demethylase genes but that overall, surprisingly, global methylation level decreases (Grzybkowska et al., 2018). When it comes to global methylation, it remains difficult to clarify the highly complex regulation of DNA methylation mechanisms during SE. However, the authors show that in SE cultures of a mutant with non-functional DRMs (*drm1 drm2*) and a triple mutant with non-functional DRMs and CMT3 (*drm1 drm2 cmt3*) SE-related genes of the *LEAFY COTYLEDON* (*LEC*) transcription factor family, *LEC1*, *LEC2* (Lotan et al., 1998; Harada, 2001; Gaj et al., 2005; Stone et al., 2008; Wójcikowska et al., 2013) and *BABYBOOM (BBM;* Boutilier et al., 2002; Casson et al., 2005) are significantly upregulated (Grzybkowska et al., 2018), which indicates that these same genes could be differentially methylated genes during SE, a hypothesis which remains to be tested in the future. In embryogenic culture of *Daucus carota*, promoters of *LEC1* and *WUSCHEL (WUS)* are hypomethylated (Shibukawa et al., 2009). Similarly, promoters of *SOMATIC EMBRYOGENESIS RECEPTOR KINASE* (*SERK*; Schmidt et al., 1997; Hecht et al., 2001), *LEC2*, and *WUS* are hypomethylated in embryogenic tissue of *Boesenbergia rotunda* (Karim et al., 2018). In addition, a recent epigenome-wide study of nine different developmental stages of SE in soybean revealed an early wave of hypermethylation, especially in the CHH context. This was linked to auxin treatment and increased RdDM activity during induction and early SE (Ji et al., 2019).

## Defects in Reproductive Development of DNA Methylation Mutants

DNA methylation mutants of *Arabidopsis thaliana* have been invaluable for exploration of mechanisms which underlie the activity of specific DNA methylation pathways during ZE and SE. First, DNA methylation mechanisms involve numerous proteins and different combinations of their mutations lead to different phenotypic characteristics, from those evident during haploid reproductive stages to those which manifest during embryogenesis. For a comprehensive list of mutations and the associated phenotypes, see Supplementary Table 1. Abolition of different DNA methylation mechanisms by loss-of-function mutations causes temporally specific phenotypes, affecting different stages of reproductive development. For instance, the loss of function of both RdDM methyltransferases, DRM1 and DRM2 (*drm1 drm2*), causes an aberrant female gametophyte, while loss of function of MET1 and CMT3 results in aberrant embryos (Jullien et al., 2012).

**FIGURE 2:**
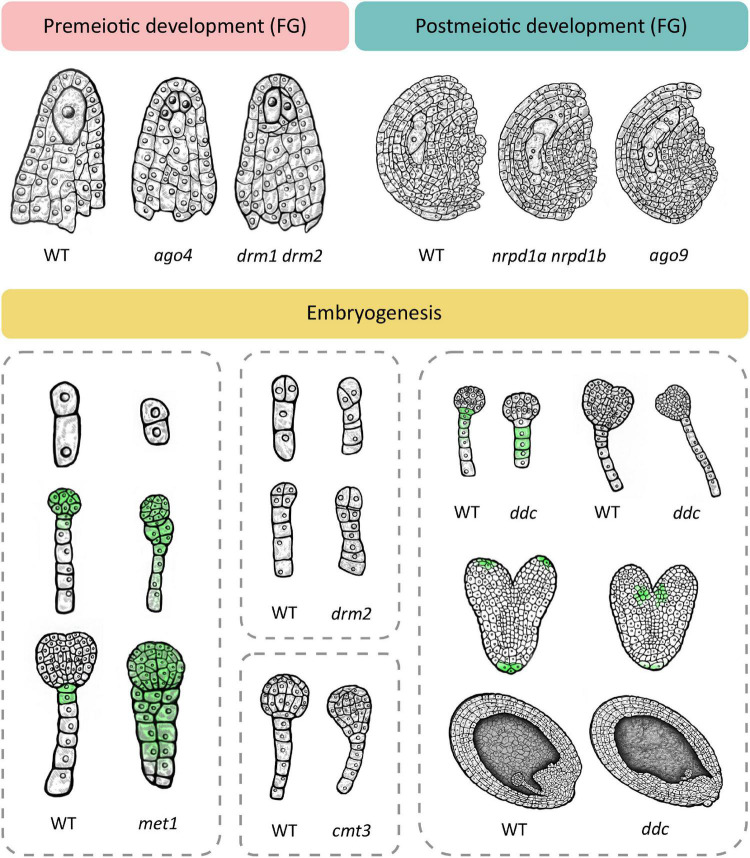
Loss of function of different methylation mechanisms leads to several dominant phenotypes at the premeiotic and postmeiotic stage and during embryogenesis. Premeiotic development. In wild type, one cell of the ovule is specified as the megaspore mother cell (MMC) which divides meiotically to give rise to the female gametophyte (FG). Several mutants with non-functional RdDM develop multiple MMC-like cells in premeiotic ovules, exemplified here in *ago4* and *drm1 drm2*. Postmeiotic development. The wild type megaspore divides mitotically to produce a female gametophyte. RdDM mutants such as *ago9* and the double *nrpd1 nrpd2* mutant exhibit two female gametophytes in postmeiotic ovules. Embryogenesis. Wild type embryogenesis begins with zygote elongation, asymmetrical division and subsequent formation of embryo and suspensor. In the *met1* mutant with non-functional MET1, the zygote remains short and divides symmetrically **(top)** and longitudinal divisions in the suspensor lead to unclear demarcation of the embryo-suspensor border **(middle and bottom)**. Additionally, auxin transport is disturbed which leads to even distribution of auxin throughout the embryo (green). The *cmt3* mutant with non-functional CMT3 also shows unclear demarcation of the embryo-suspensor border in early globular stage. Loss of function of RdDM leads to similar aberrations. The *drm2* mutant shows disturbed patterns of cell divisions in the early embryo in both the suspensor **(top and bottom)** and the embryo proper **(bottom)**. The triple *drm1 drm2 cmt3* mutant (*ddc*) exhibits a reduced number of suspensor cells at the globular stage with a hypophysis devoid of auxin signal **(top left)** and a longer suspensor at early heart stage **(top right)**. At the heart stage, auxin maximums appear basally from cotyledons **(middle)**. Wild type embryos are positioned in parallel with the top–bottom axis of the ovule. In a portion of *ddc* mutant plants, embryos are positioned perpendicular to the axis, and the endosperm is histologically disorganized **(bottom)**. This schematic image was created based on phenotypes described in relevant scientific articles. For details and references, see Supplementary Table 1.

Phenotypic changes related to premeiotic development can be observed during cell fate specification of the megaspore mother cell (MMC). In wild type Arabidopsis, one cell of the hypodermal ovule layer is specified as the MMC. In the double *drm1 drm2* mutant, multiple cells become specified as the MMC, resulting in multiple precursors of the female gametophyte (Mendes et al., 2020; Figure 2). Here, loss of DRM function causes upregulation of the *SPOROCYTELESS/NOZZLE* (*SPL/NZZ*) transcript encoding a protein involved in balancing the reproductive cell fate establishment in the premeiotic ovule (Mendes et al., 2020). A similar phenotype develops in mutants of the *AGO4*, *AGO6*, *AGO8* and *AGO9* genes (shown for *AGO4* in Figure 2), and depending on the mutated gene, the number of MMCs varies, from two to four (Hernández-Lagana et al., 2016). Multiple MMC-like cells are also caused by loss-of-function mutations of genes encoding proteins involved in the PolIV arm of RdDM, such as the aforementioned polymerase RDR2 (*rdr2*) which produces double-stranded siRNAs, its ortholog RDR6 (*rdr6*) which acts in non-canonical RdDM, an RNA-binding protein called SUPPRESSOR OF GENE SILENCING 3 (*sgs3*) and the siRNA-processing protein DCL3 (*dcl3*) (Olmedo-Monfil et al., 2010). A similar phenotype is also found in a double mutant in which both NRPD1a and NRPD1b (also known as NRPE1), the respective largest subunits of PolIV and PolV are mutated (*nrpd1a nrpd1b*) and both polymerases are non-functional (Olmedo-Monfil et al., 2010). It should be noted here that loss of function of newly discovered ET demethylases decreases the *SPL/NZZ* expression (Tedeschi et al., 2019) suggesting the possible balancing effects of RdDM methylation and ET-specific demethylation during plant reproduction.

Besides exhibiting a premeiotic phenotype, *ago9, rdr2*, *dcl3* and the *nrpd1a nrpd1b* double mutant are additionally affected in postmeiotic development, with noted formation of multiple female gametophytes (shown for *nrpd1a nrpd1b* and *ago9* in Figure 2). In some cases, two developing gametophytes are separated by several somatic cells, indicating that they originated from non-sister cells, of which one had to be of somatic origin (Olmedo-Monfil et al., 2010). This phenomenon could serve as an illustration of the potency of epigenetic mechanisms in regulating development and even establishing a novel trajectory of development from unlikely origins, as described for SE. In the aforementioned mutants with non-functional RdDM, methyltransferase MET1 is functional but it does not compensate for the lack of RdDM, possibly due to low expression of *MET1* (Jullien et al., 2012) or the functional limitations of MET1 activity, i.e., its dependence on previous methylation and specificity for the CG context.

Deficiencies in RdDM and other DNA methylation mechanisms also cause aberrations during embryonic development. Interestingly, the loss-of-function MET1 mutant (*met1*), displays a wide array of successive phenotypes (Xiao et al., 2006; Figure 2) which first manifest during the elongation and asymmetric division of the zygote and continue later with abnormalities in numbers and planes of cell division throughout embryogenesis as well as delays in embryo development. According to Xiao et al. (2006), loss of MET1 directly or indirectly affects transcription of genes that regulate cell identity during early embryogenesis. Specifically, it causes downregulation of *WOX2* and *WOX8*, upregulation of *YDA* and altered expression pattern of *PIN1*, which becomes evenly distributed throughout the entire embryo, in stark contrast to its usual accumulation in the apical cell-derived regions. Concurrently, auxin becomes evenly distributed in both the apical and basal cell-derived regions, which hinders the establishment of the auxin maximum, possibly accounting for the lack of demarcation between embryo and suspensor (Figure 2). The authors postulate that hypomethylation is the most probable cause of phenotypic defects in the *met1* mutant. They also suggest the possibility of compensation for loss of CG-specific MET1 through induced activation of other methylation mechanisms which could then cause ectopic hypermethylation on specific positions and result in further developmental aberrations (Xiao et al., 2006).

Loss of function of the non-CG-specific methyltransferase CMT3 (*cmt3*) leads to aberrations in later stages of embryogenesis, with a lack of clear demarcation between the embryo and suspensor due to longitudinal cell divisions in the suspensor (Figure 2). The double *met1 cmt3* mutant embryos display similar aberrations but with more dramatic effects on embryo development, seed viability and plant development (Xiao et al., 2006).

Unlike MET1 and CMT3, *de novo* methyltransferase DRM2 can induce DNA methylation in all three sequence contexts (Chan et al., 2005). Ingouff et al. (2017) show that the *drm2* mutant suffers a global loss of maternally provided CHH methylome in the egg cell, causing abnormal patterning and division plane defects in the early embryo (Figure 2). Furthermore, the triple *ddc* mutant, in which DRM1, DRM2 and CMT3 are non-functional, shows various phenotypic aberrations during embryogenesis, which has been linked to an impaired auxin pathway (Forgione et al., 2019; Figure 2). In the early embryo stage, the *ddc* mutant exhibits a reduced number of suspensor cells and a delayed suspensor development. In the globular stage, suspensor cell proliferation is arrested, resulting in a shorter suspensor with a hypophysis devoid of auxin signal, while increased proliferation and a more elongated suspensor marks the young heart embryo stage. When the embryo reaches heart stage, auxin maximums appear basally from cotyledons, contrary to the usual accumulation of auxin in the apical regions of the cotyledons (Figure 2). Finally, aberrations in the embryo are combined with disordered histological organization of the endosperm (Figure 2). Interestingly, this aberration reminds of a phenotype described for the *yda* mutant, where embryos are positioned perpendicular to the top-bottom axis of the ovule, as if lying on their sides (Lukowitz et al., 2004). The leaf of the *ddc* mutant is marked by increased expression of genes involved in the auxin biosynthesis pathway, namely *YUC2* and *TAA1*, and while *TAA1* was not differentially methylated, the authors report significant demethylation in the promoter region of *YUC2* (Forgione et al., 2019). Although gene expression and methylation levels of auxin-related genes have not been examined in *ddc* embryos (Forgione et al., 2019), the results obtained in leaf tissues combined with described auxin-related embryo aberrations serve as a novel link between *de novo* DNA methylation and the role of auxin pathways in embryonic development, which remains to be further explored in the future.

In mammals, loss-of-function mutation of DNA methyltransferase Dnmt1 causes an embryo lethal phenotype (Li et al., 1992), a dramatic effect which does not occur in plants, including Arabidopsis, when either of their three major methyltransferases is mutated. On the other hand, a number of methylation mutants of investigated plant species were shown to be either lethal at some point during development, hypomorphic, or depleted in multiple methylation contexts (Domb et al., 2020). To date, an Arabidopsis mutant with a complete loss of all DNA methylation has not been described, as zero-methylation state is most likely lethal. The existence of single mutants, however, suggests redundancy between mechanisms, additionally supported by the fact that mutations affecting more than one methylation mechanism lead to more pronounced developmental aberrations (Xiao et al., 2006). Interestingly, single-mechanism mutations lead to temporally specific phenotypes, indicating activity shaped by developmental needs. RdDM is particularly interesting in this aspect as it could naturally serve as a potent mechanism in not only substituting for missing methylation marks, but also in establishing novel methylation patterns in response to various internal and external cues. The RdDM pathway is comprised of numerous components, not all of which are indispensable for DNA methylation to occur. The highest level of functional promiscuity has been ascribed to DMS3, a protein which recruits PolV to the genome, and which seems to perform this role even when most other RdDM components have been mutated (Gallego-Bartolomé et al., 2019). The research of RdDM seems to be marked by exceptions, rather than rules, which could point to the pathway’s highly versatile roles, at least some of which could be linked to embryogenesis, including a specific role of auxin dynamics in regulating embryonic development. Clarification of the role of RdDM in these processes could be aided by identification of genes directly regulated by RdDM-mediated DNA methylation during embryogenesis. In the following section, we bring an overview of genomic regions which are potential targets of the PolV polymerase, a component of RdDM which determines the future methylation site, and analysis of loci specifically linked to auxin dynamics, reproductive development and embryogenesis.

## Determination of Genomic Loci Targeted by RNA-Directed DNA Methylation

The chromatin association profile of NRPE1 (the largest subunit of PolV) in *Arabidopsis thaliana* Col-0 flowers is published by Liu et al. (2018). To determine specific genes potentially regulated by RdDM, read filtering, mapping, peak calling and peak annotation was performed to retain only the peaks associated with 1142 genes categorized into 79 gene ontologies (GO) related to auxin metabolism, reproductive development, and zygotic and somatic embryogenesis (for a complete list of GOs, refer to Supplementary Table 2). Finally, of the 441 remaining peaks, we retained peaks with fold change greater than 2.0, *p*-value less than 10^-12 and which were positioned up to 3000 bp upstream from the associated gene, resulting in 224 peaks in total (Supplementary Table 3). Following selection, most of the auxin metabolism genes with known roles in SE or ZE mentioned earlier were found as targets of PolV. Namely, the list contained genes involved in the biosynthesis of auxin (*TAA1, TAR1, TAR2, YUC2, YUC5, YUC10, YUC1, LEC2*), in the regulation of directed auxin transport (*PIN3, PIN4, PIN7*) and genes encoding auxin response factors (*ARF1, ARF2, ARF8*) and AUX/IAA inhibitors (*IAA6, IAA8, IAA14, IAA18, IAA27*). It was previously shown that *YUC2* is hypomethylated in the *dcc* mutant, indicating a role of RdDM, possibly in combination with CMT3, while *PIN1*, *PIN3*, *PIN4*, and *PIN7* have been suggested as potential targets due to their variable expression in the *ddc* mutant (Forgione et al., 2019). In addition, the *WOX8* gene encoding a protein involved in establishment of apical-basal axis in the young embryo was also identified as a potential PolV target. Although it was previously shown that regulation of the *WOX2*/*WOX8* pair depends on MET1 (Xiao et al., 2006), the connection with RdDM indicates the redundancy of this pathway in the *WOX2*/*WOX8* gene expression regulation.

One of our additional criteria for gene selection was position of the peak up to 3000 bp upstream from the TSS of an associated gene. Zhong et al. (2012) show that PolV binds to promoters and that the loss of its largest subunit (NRPE1) leads to an increase in expression of genes located near the PolV binding site. Specifically, when PolV is non-functional, the effect of its loss on gene expression, i.e., upregulation, is higher for genes which have the PolV binding site closer to the TSS (Zhong et al., 2012). Therefore, we selected genes with up to 50 bp distance between the peak and the TSS to generate a list of genes most likely to be regulated by RdDM. This selection resulted in a list of 22 genes (Table 2), among which only *SIR3* is functionally related to stress response. The remaining 21 genes are directly or indirectly related to reproductive development and their loss of function leads to aberrations in megaspore development, formation of supernumerary egg cells, zygotes or embryos and disturbances in auxin metabolism, transport or effects (for references see Table 2). Additionally, some of these genes affect embryogenesis through regulation of transcription, posttranscriptional regulation, proteasomal degradation, cell-to-cell signalization, t-RNA splicing, flavonoid biosynthesis, and biogenesis of multifunctional iron–sulfur clusters (for references see Table 2). Interestingly, out of 22 genes on the list, six belong to Early Culture Abundant 1 (ECA1) gametogenesis-related family, which is one of the three largest families encoding small cysteine-rich proteins, many of which are expressed during reproductive development (reviewed in Sprunck et al., 2014). Members of this family were first described in barley, where HvECA1 is responsible for stress-induced switch from gametophytic pathway to embryogenic route (Vrinten et al., 1999). Functional characterization of HvECA1 resulted in discovery of a significant number of similar CRPs in egg cell transcriptomes of different flowering plants. In Arabidopsis, there are 124 genes of ECA1 gametogenesis-related family (Sprunck et al., 2014). The best described protein candidate, EGG CELL 1 (EC1), is secreted from the egg cell and responsible for sperm cell activation to gain competence for gamete fusion, which indicates that it is essential for the reproductive phase of development (Sprunck et al., 2012). Besides egg cell-specific genes, a significant number of ECAs are expressed in synergids under control of the synergide-specific MYB98 transcription factor (Jones-Rhoades et al., 2007). Sprunck et al. (2014) argue that members of this family potentially partake in different processes related to reproductive development, including androgenesis, as occurs in barley (Sprunck et al., 2014). Our overview of PolV-bound genomic loci indicates ECA1 gametogenesis-related proteins as interesting targets for further research of RdDM roles in reproductive development. Interestingly, genes encoding ECA1 gametogenesis-related proteins have an unusual transposon-like pattern of methylation, in which RdDM mediates gene body methylation in CG, CHG and CHH contexts. This type of methylation is generally linked to expression downregulation in vegetative tissues and is usually low in synergids, in which many CRP genes are expressed (You et al., 2012). Therefore, ECA1 gametogenesis-related family could be additionally used to study the role of RdDM in transition between the reproductive and vegetative stage.

**TABLE 2:** Potential PolV binding sites.

Gene	Locus	Position relative to TSS/gene	Protein function	Development/phenotype	References
*RIE1*	AT2G01735	0/overlap with start	E3 ubiquitin ligase	Seed development/Arrest at globular stage	Xu and Li, 2003
*ADA2B*	AT4G16420	0/overlap with start	Transcriptional adapter	Pleiotropic/Auxin overproducing mutant-like phenotype	Vlachonasios et al., 2003
*ZAR1*	AT2G01210	0/overlap with start	Receptor protein kinase-like	Zygote asymmetric division and daughter cell fate	Yu et al., 2016
*AGL23*	AT1G65360	0/overlap with start	Agamous-like MADS-box	Female gametophyte and chloroplast development in embryo/developmental arrest at the megaspore stage	Colombo et al., 2008
*SIR3*	AT1G16540	0/overlap with start	Molybdenum cofactor sulfurase (LOS5) (ABA3)	Conversion of ABA-aldehyde to ABA/Modulates cold and osmotic stress responsive genes	Xiong et al., 2001
ECA1 gametogenesis related family	AT2G24205	0/overlap with entire gene	ECA1 gametogenesis related family protein	Flowering plant reproduction/not tested	Sprunck et al., 2014
*EXPB2*	AT1G65680	0/overlap with start	Putative expansin-B2	Unidimensional cell growth, expressed in reproductive tissues of maize/Drought resistance	Wu et al., 2001; Ezquer et al., 2020
ECA1 gametogenesis related family	AT5G44495	0/overlap with entire gene	Small signaling CRP	Flowering plant reproduction/not tested	Sprunck et al., 2014
ECA1 gametogenesis related family	AT5G60964	0/overlap with entire gene	Small signaling CRP	Flowering plant reproduction/not tested	Sprunck et al., 2014
SAUR-like auxin responsive family	AT5G42410	0/overlap with start	SAUR43	Substrate of RDR1/Not expressed in rdr1 mutants	Hua et al., 2021
EMB1691	AT4G09980	0/overlap with start	Methyltransferase B	N6-adenosine methylation of mRNA/mRNA modification, splicing, metabolism	Muñoz-Nortes et al., 2017; Meinke, 2019
ECA1 gametogenesis related family	AT5G60945	0/overlap with entire gene	Small signaling CRP	Flowering plant reproduction/not tested	Sprunck et al., 2014
ECA1 gametogenesis related family	AT5G42895	0/overlap with entire gene	Small signaling CRP	Flowering plant reproduction/not tested	Sprunck et al., 2014
*PIN4*	AT2G01420	0/overlap with start	Auxin efflux carrier component	Maintenance of embryonic auxin gradients/Root pattering	Friml et al., 2002
*SEN1*	AT3G45590	2/upstream	DNA helicase	tRNA splicing in the initiation of zygote division/zygote-lethal	Yang et al., 2017
*LIS*	AT2G41500	24/upstream	a protein with seven WD40 repeats	Prevents accessory cells from adopting gametic cell fate/supernumerary egg cells	Groß-Hardt et al., 2007
ECA1 gametogenesis related family	AT2G27315	28/overlap with end	Small signaling CRP	Flowering plant reproduction/not tested	Sprunck et al., 2014
EMB1796	AT3G49240	35/upstream	Pentatricopeptide repeat-containing protein	Posttranscriptional RNA editing/Embryo lethality	Guillaumot et al., 2017
NAC081	AT5G08790	37/upstream	NAC family transcription factor	Regulates *NIT2* gene involved in auxin biosynthesis/Reduced sensitivity to indole-3-acetonitrile	Huh et al., 2012
CYP75B1/TT7	AT5G07990	40/upstream	Flavonoid-30-hydroxylase	Flavonoid biosynthetic pathway/Modulated auxin transport	Peer and Murphy, 2007
*ABCI7* (SufD)	AT1G32500	48/upstream	ATP-binding cassette (ABC) proteins	Fe-S cluster biogenesis, housekeeping functions in embryogenesis/Globular stage lethality	Xu and Møller, 2011
*PIN7*	AT1G23080	50/inside gene	Auxin efflux carrier component 7	Setting up the apical-basal axis in the embryo/Failed to establish the apical–basal auxin gradient	Friml et al., 2003; Robert et al., 2013

*ChIP-seq data obtained with anti-NRPE1 antibody in Col-0 flower tissues published in Liu et al. (2018) were reanalyzed with a focus on targets involved in reproductive development, embryogenesis and auxin metabolism. Genes with associated peaks positioned up to 50 bp upstream from the TSS were selected from the 224 peaks listed in Supplementary Table 3.*

## Concluding Remarks and Perspectives

There are still many aspects of plant embryogenesis that are not fully understood, especially at its onset. How is the reprogramming of the transcriptome and DNA methylome at the onset of embryogenesis controlled and what are the signals that direct or redirect the zygote or a somatic cell into a state of embryogenic competence? There is substantial evidence linking RdDM to gametophyte development and embryogenesis, but the exact mechanisms through which RdDM could regulate gene expression prior to and at the onset of plant embryogenesis remains to be elucidated. Here, we propose a list of genes presumably targeted by PolV, which could serve as a pool of gene candidates for future research of the roles of RdDM in reproductive development and embryogenesis, as well as the mechanisms by which auxin dynamic might shape these processes. Different components of the RdDM pathway certainly play their own distinct roles in this process. For instance, members of the AGO4 clade, consisting of AGO4, AGO6, and AGO9, all participate in the RdDM pathway but functionally diverge in terms of their ability to promote short RNA accumulation and DNA methylation, and this distinction is present even when different AGOs bind the same short RNAs (Havecker et al., 2010). At least in part, the difference in AGO function could be attributed to their distinct expression profiles (Havecker et al., 2010), with AGO9 primarily expressed in female gametes, where it has a role in TE silencing (Olmedo-Monfil et al., 2010). The specificity of individual components of RdDM for distinct tissues and even cell types could indicate the existence of specialized branches of RdDM, assembled according to different biological requirements and possibly consisting of undiscovered and highly specialized associated factors. In the future, it would be interesting to compare the siRNA profile of AGO9 with the PolV-bound genome sites, and potentially retrieve a set of genes presumably regulated by RdDM in a tissue-specific manner, with functions related to female gametophyte development. Clarification of the RdDM mechanism at the onset of embryogenesis is also of practical value, as it could open the door to an applicative function combining DNA methylation-based techniques with SE- mediated propagation. Treatment with epigenetic regulators that induce global demethylation, such as 5-azacytidine, was shown to be beneficial in plant breeding (Kondo et al., 2006), showing that loss of methylation can be a significant source of variation, with potentially favorable effects. On the other hand, the application of CRISPR/Cas technology to edit epigenetic marks at specific loci (McDonald et al., 2016; Papikian et al., 2019) and to consequently modulate gene expression, may lead to more precise and predictable breeding (Mercé et al., 2020), especially if we take into account that epigenetic marks are heritable through at least a few generations (Papikian et al., 2019).

## Author Contributions

DLL developed the idea. LM carried out the bioinformatics and determination of RdDM genomic loci. MT and AŠ performed the mutant manuscript analysis and prepared the illustrations. DLL and AŠ drafted and wrote most of the manuscript while MT, NB, MJ, and TV participated in writing. All the authors contributed to the article and approved the submitted version.

## Conflict of Interest

The authors declare that the research was conducted in the absence of any commercial or financial relationships that could be construed as a potential conflict of interest.

## Publisher’s Note

All claims expressed in this article are solely those of the authors and do not necessarily represent those of their affiliated organizations, or those of the publisher, the editors and the reviewers. Any product that may be evaluated in this article, or claim that may be made by its manufacturer, is not guaranteed or endorsed by the publisher.
