# Supplementary material for: Taking the Wheel – de novo DNA Methylation as a Driving Force of Plant Embryonic Development
Source: Front Plant Sci. 2021 Oct 29;12:764999. doi: 10.3389/fpls.2021.764999 (PMC8585777; doi:10.3389/fpls.2021.764999)
Supplement: Supplementary file 1 [file Table_1.docx]

**TABLE S1.** Phenotypes in reproductive development of different DNA methylation defective mutants. Mutants related to the main components of the canonical RdDM pathway are bolded. When information was available, genes that might be responsible for observed phenotype/s are given.

| **Mutant/s** | **Phenotype** | **Reference** |
| --- | --- | --- |
| *met1* | Abnormal integument growth and campylotropy; altered number and planes of cell division required for generating the embryo proper, apical-basal axis, cotyledons, and meristems; no demarcation between embryo and suspensor (many longitudinal cell divisions in the suspensor); delayed embryo development.  A downregulation of *WOX2* and *WOX8*, upregulation of *YDA*, and altered expression pattern and miss-localization of *PIN1* may be implicated as the cause of the observed phenotype. | Xiao et al., 2006 |
| *cmt3* | Abnormal cell divisions in the transition zone between the suspensor and embryo at 2-cell stage; no clear demarcation between embryo and suspensor at 8 to 16-cell and 32-cell embryo stages (excess longitudinal cell divisions in the suspensor); abnormalities in planes of cell division in embryo proper. | Pillot et al., 2010 |
| ***drm2*** | Abnormal early embryo patterning; aberrant divisions in either the embryo proper or the suspensor domain. | Inguoff et al., 2017 |
| ***drm1 drm2*** *cmt3*  ***(ddc)*** | Reduced number of suspensor cells (arrest of cell proliferation) at globular stage; longer suspensor with more cells (postponed cell proliferation) at early heart stage; disordered histological organization of the endosperm; alteration in the establishment of auxin gradients. | Forgione et al., 2019 |
| ***ago4,*** *ago6*, *ago8*, *ago9*,  ***dcl3****,* ***drm1 drm2****,*  ***nrpd1a nrpd1b***,  ***rdr2***, *rdr6,*  *sgs3* | Multiple megaspore mother cell-like cells in premeiotic ovules.  A transcriptional upregulation of SPOROCYTELESS/NOZZLE (*SPL/NZZ*) in *ago9, rdr6* and *drm1 drm2* was proposed as the cause of the observed phenotype. | Hernández-Lagana et al., 2016; Mendes et al., 2020; Olmedo-Monfil et al., 2010 |
| *ago9,* ***dcl3****,*  ***nrpd1a nrpd1b***,  ***rdr2***, *rdr6* | Post meiotic ovule with two developing female gametophytes. | Olmedo-Monfil et al., 2010 |
